# Taiwan Society of Colon and Rectum Surgeons (TSCRS) Consensus for Anti-Inflammatory Nutritional Intervention in Colorectal Cancer

**DOI:** 10.3389/fonc.2021.819742

**Published:** 2022-01-17

**Authors:** Cheng-Jen Ma, Wan-Hsiang Hu, Meng-Chuan Huang, Jy-Ming Chiang, Pao-Shiu Hsieh, Huann-Sheng Wang, Chien-Ling Chiang, Hui-Min Hsieh, Chou-Chen Chen, Jaw-Yuan Wang

**Affiliations:** ^1^ Division of Colorectal Surgery, Department of Surgery, Kaohsiung Medical University Hospital, Kaohsiung Medical University, Kaohsiung, Taiwan; ^2^ Division of General and Digestive Surgery, Department of Surgery, Kaohsiung Medical University Hospital, Kaohsiung Medical University, Kaohsiung, Taiwan; ^3^ Division of Colorectal Surgery, Department of Surgery, Chang Gung Memorial Hospital–Kaohsiung, Kaohsiung, Taiwan; ^4^ Division of Nutrition and Dietetics, Kaohsiung Medical University Hospital, Kaohsiung Medical University, Kaohsiung, Taiwan; ^5^ Division of Colon and Rectal Surgery, Department of Surgery, Chang Gung Memorial Hospital–Linkou, Taoyuan, Taiwan; ^6^ Division of Colon & Rectal Surgery, Department of Surgery, Taipei Veterans General Hospital, Taipei, Taiwan; ^7^ Division of Nutrition, Chang Gung Memorial Hospital–Linkou, Taoyuan, Taiwan; ^8^ Division of Nutrition, Taichung Veterans General Hospital, Taichung, Taiwan; ^9^ Division of Colorectal Surgery, Department of Surgery, Taichung Veterans General Hospital, Taichung, Taiwan; ^10^ Department of Surgery, Faculty of Medicine, College of Medicine, Kaohsiung Medical University, Kaohsiung, Taiwan; ^11^ Graduate Institute of Clinical Medicine, College of Medicine, Kaohsiung Medical University, Kaohsiung, Taiwan; ^12^ Graduate Institute of Medicine, College of Medicine, Kaohsiung Medical University, Kaohsiung, Taiwan; ^13^ Center for Cancer Research, Kaohsiung Medical University, Kaohsiung, Taiwan; ^14^ Center for Liquid Biopsy and Cohort Research, Kaohsiung Medical University, Kaohsiung, Taiwan; ^15^ Clinical Pharmacogenomics and Pharmacoproteinomics, College of Pharmacy, Taipei Medical University, Taipei, Taiwan; ^16^ Pingtung Hospital, Ministry of Health and Welfare, Pingtung, Taiwan

**Keywords:** anti-inflammation, systemic inflammatory response, neutrophil-to-lymphocyte ratio, malnutrition, colorectal cancer

## Abstract

Malnutrition and systemic inflammatory response (SIR) frequently occur in patients with colorectal cancer (CRC) and are associated with poor prognosis. Anti-inflammatory nutritional intervention is not only a way to restore the malnourished status but also modulate SIR. Nine experts, including colorectal surgeons, physicians and dieticians from 5 hospitals geographically distributed in Taiwan, attended the consensus meeting in Taiwan Society of Colon and Rectum Surgeons for a 3-round discussion and achieved the consensus based on a systematic literature review of clinical studies and published guidelines. The consensus recommends that assessment of nutritional risk and SIR should be performed before and after CRC treatment and appropriate nutritional and/or anti-inflammatory intervention should be adapted and provided accordingly.

## Introduction

Malnutrition is frequently encountered in patients with cancer and varied from 34% to 71% (1–5). Cancer is a condition that increases metabolism, which raises the body’s needs for energy and protein. Moreover, symptoms such as anorexia, nausea, vomiting, early satiety, pain, bleeding, and bowel obstruction that result from cancer itself reduce food intake, and this phenomenon is especially observed in gastrointestinal cancer. Toxicities of chemotherapy and radiotherapy (e.g., mucositis, fatigue, constipation and diarrhea) as well as surgical outcomes further impair oral feeding. In addition, mental reactions to disease and treatments, which include depression, psychological stress and decreased physical activities also influence the appetite. A long-term lack of enteral stimulation by dietary intake results in the reduction of intestinal mucosal cell proliferation. Therefore, intestinal mucosal atrophy and collapse of intestinal mucosal barrier occur and cause malabsorption (6), which generates a vicious circle along with malnutrition.

During cancer progression, proinflammatory cytokines produced by cancer cells and host immune response to both tumors and cancer treatments result in systemic inflammatory response (SIR) (7, 8), which provokes metabolism. SIR affects the brain, muscle, liver, and fat function (9), leading to anorexia (10), muscle wasting (11), suppressed anticancer drug clearance (12), and depletion of fat (13), respectively. In turn, an imbalance between anabolism and catabolism worsens the nutritional status in cancer patients and results in a depletion of the body’s energy reserves. In the context of skeletal mass decline (i.e. sarcopenia), malnutrition has been associated with an increased risk of both surgery complications and chemotherapy adverse events (14–16). Consequently, dosage of anticancer drugs may be reduced, delayed or interrupted compared to the treatment recommendation, and all of these are associated with poor survival (17–21). Moreover, sarcopenia further limits physical activity, which has adverse impacts on quality of life (QoL). Studies have demonstrated SIR, which is indicated by a high neutrophil-lymphocyte ratio (NLR) (22–26), an elevated C-reactive protein (CRP) (22, 23, 27–33) and Glasgow Prognostic Score (GPS) (i.e. the combination of elevated CRP and hypoalbuminemia) (34–40), is associated with a poor prognosis in cancer patients.

## Methods

The literature in PubMed was searched for eligible studies published using the keywords “colorectal neoplasms” or “colorectal cancer,” “neoplasms” or “cancer,” “nutrition,” “malnutrition,” and “inflammation.” Clinical trials, non-controlled trials, and descriptive studies such as cohort studies, case series, and case reports published between January 1990 and December 2020 were retained. Published guidelines and consensus documents were also retrieved. The highest level of evidence available according to the systemic review of literature was assigned to each statement to categorize the quality of each statement to facilitate decision-making for panel members. Levels of evidence ranged from level 1 (randomized controlled trials) to level 5 (expert opinion) based on Oxford Centre for Evidence-Based Medicine.

**Figure 1:**
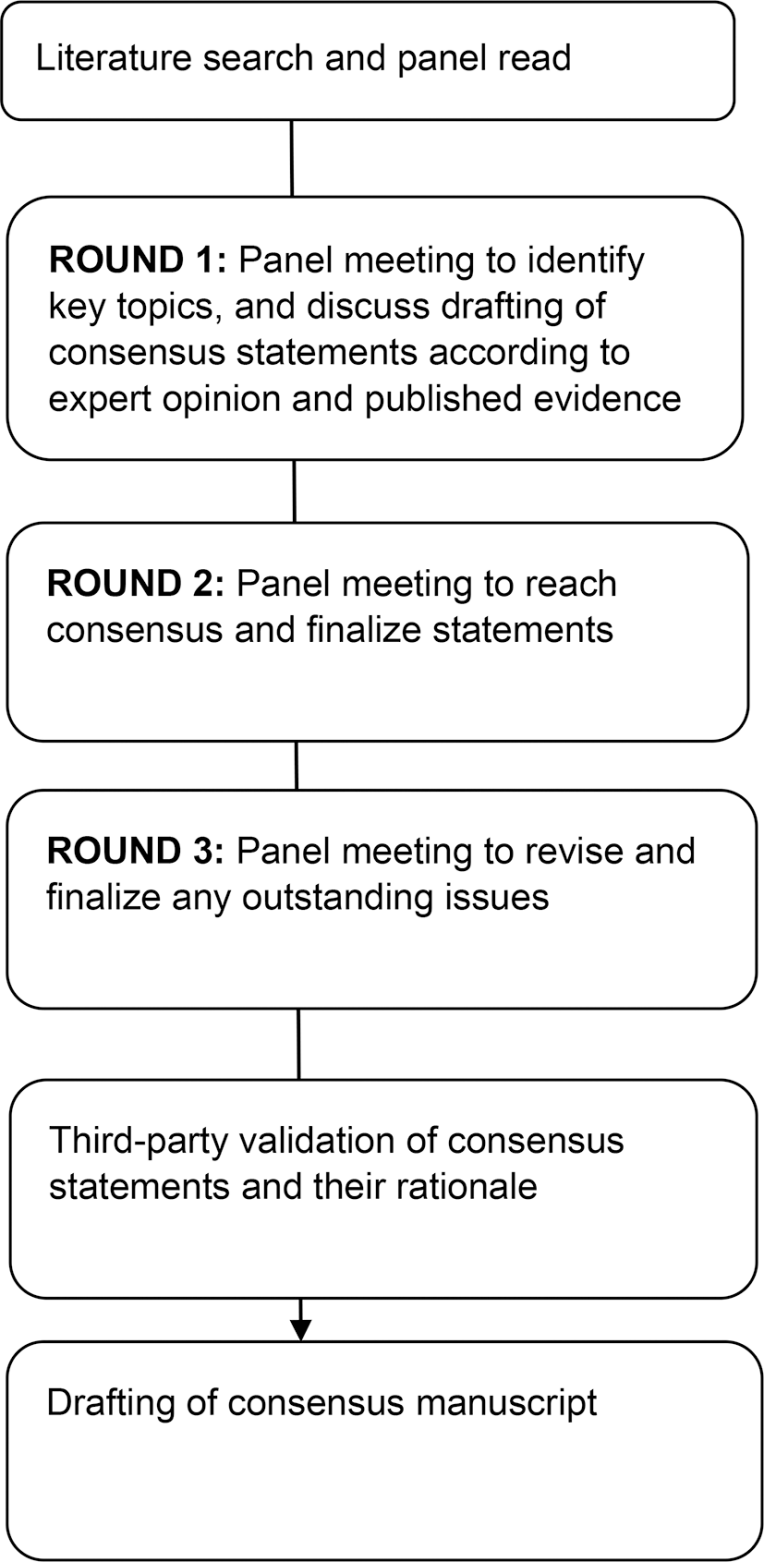
CONSORT flow diagram of consensus forming process.

A modified Delphi process was adopted. The Taiwan Society of Colon and Rectum Surgeons (TSCRS) invited a group of 9 experts (colorectal surgeons, oncologists, and dieticians) from five hospitals geographically spread over Taiwan to review and discuss the literature face-to-face over three rounds (**Figure 1**). The panel members drafted consensus statements and the panel voted by showing hands for each statement and the consensus was formed if more than two-thirds of the panel members agreed.

### Round 1

The panel first identified 3 key topics of inflammation in colorectal cancer (CRC) and further refined their search for, review of, and discussion of the evidence to address clinical questions focusing on (1): markers of inflammatory status in CRC (2); the prognostic value of inflammatory markers for CRC and NLR, CRP, and GPS were accepted; and (3) anti-inflammatory nutritional interventions in CRC and long-chain omega-3 polyunsaturated fatty acids (n-3 PUFAs) and glutamine were accepted.

### Round 2

In round 2, discussion for specific inflammatory marker and anti-inflammatory nutritional intervention was launched. As the result, NLR and n-3 PUFAs were accepted into the final guideline document.

### Round 3

Panel members were encouraged to discuss the statements in the final guideline document to determine the cut-off value of NLR and to revise and finalize any outstanding issues. In the end, the threshold for NLR ≥3 was accepted and post-treatment anti-inflammatory nutritional intervention was added.

The questions, statements, and their rationale were presented to a third-party panel composed of 2 colorectal surgeons, 2 oncologists and 2 dieticians for validation. Each question was discussed by the third party to eliminate bias and again, statements has to be voted and agreed with if more than two-thirds of the members of the third-party panel. The final statements were based on the level of evidence and the level of consensus. This document is an independent report of the panel and is not a policy statement of the TSCRS.

## Prognostic Value of NLR in Patients with Cancer

NLR is one of the most evaluated inflammatory markers that are used as a predictor for outcomes of treatments in various cancer types. According to a review article published in 2018, which reviewed 36 trials containing data on 40,354 patients, CRC was the most common type with 10 trials containing data on 27,438 patients until January 2018, and NLR/derived NLR was assessed in 33 trials with data on 39,313 patients (41). NLR is calculated by dividing the neutrophil count by the lymphocyte count and can be used as an economic and simple inflammatory marker to measure SIR, since blood count is one of the initial routine examinations for patients in clinical practice (42). Neutrophils and lymphocytes are one of the first inflammatory cells that respond to SIR. Neutrophils, together with cancer cells, secret proinflammatory cytokines, chemokines and growth factors, where neutrophils are a source and a target at the same time. The secreted factors include interleukin (IL)-1, IL-6, IL-8, tumor necrosis factor-alpha (TNF-α), hepatocyte growth factor (HGF) and vascular endothelial growth factor (VEGF), which in turn promote not only tumor growth but also cancer metastasis in the tumor microenvironment (43). Moreover, neutrophils can inhibit the lymphocytes’ cytotoxic activities against cancer cells, resulting in immune escape of cancer cells (43). Therefore, high NLR is associated with poor oncological outcomes.

### Prognostic Value of Pre-Treatment NLR in CRC

Studies of prognostic value of pre-treatment NLR in CRC are summarized in **Table 1**. It is revealed that not only higher NLR in CRC, adenomatous polyp and healthy individuals respectively (60–62), but also an elevated NLR positively correlates with larger tumor size, histological grade, and more advanced T, N, and TNM stage (24, 44, 45). As such, pre-operative high NLR was found significantly associated with shorter overall survival (OS), disease-free survival (DFS), and cancer-specific survival (CSS) (44–50), and this was more prominent in patients with diseases at stage III and IV (24, 51–55). In contrast, the association was controversial in early stage CRC (stage I and II). Some studies showed no significant association between NLR and stage I/II patients (24, 52), but other studies demonstrated that high pre-operative NLR significantly correlated with poor OS in stage II cancer (51), DFS in stage I/II cancer (56), or DFS and CSS in stage I cancer (57). For stage I/II diseases, the combination of NLR and other inflammatory markers may provide a more significant prognostic value. Inamoto et al. revealed that the combination of NLR and GPS stratified OS, CSS and DFS in stage I/II CRC, though this was not as pronounced as in stage III/IV CRC (48). In addition, high pre-treatment NLR predicted not only prognosis of operation, but also predicted decreased progression-free survival (PFS) and OS in CRC patients receiving neoadjuvant chemoradiotherapy (58), and OS in unresectable metastatic CRC (mCRC) patients receiving chemotherapy and biological agent (59).

**Table 1:** Studies of prognostic value of pre-treatment NLR in CRC.

Impact factor	Evidence level	Study	Sample size	Cut-off value of NLR	TNM stage	Negative impact on survival
2.7	2b	Kubo et al., 2016 (24)	823	2.1	I-IV	CSS in stage III/IV
2.9	2b	Song et al., 2017 (44)	1,744	2.0	I-IV	OS, CSS
3.1	2b	Rashtak et al., 2017 (45)	2,536	3.0	I-III	DFS, OS
4.9	2b	Li et al., 2016 (46)	5,336	2.72	I-III	DFS, OS
3.3	2b	Li et al., 2016 (47)	140	2.3	I-III	DFS, OS
2.6	2b	Inamoto et al., 2019 (48)	448	2.05	I-IV	DFS, OS, CSS
1.8	2a	Li et al., 2019 (49)	5,897 (meta-analysis)	–	–	DFS, OS, CSS, RFS
1.3	2b	Gulben et al., 2020 (50)	219	2.8	I-III	OS
3.6	2b	Choi et al., 2015 (51)	549	2.6	I-III	OS in stage II/III, RFS in stage III
3.4	2b	Kim et al., 2017 (52)	1,868	3.0	I-IV	DFS, OS in stage III/IV
3.1	2b	Giakoustidis et al., 2015 (53)	169	2.5	IV	OS
2.8	2a	Tang et al., 2016 (54)	1,685 (meta-analysis)	–	IV	OS, RFS
3.1	2b	Cruz-Ramos et al., 2019 (55)	110	3.02	IV	OS, PFS
3.4	2b	Galizia et al., 2015 (56)	276	2.36	I-II	DFS
3.1	2b	Shin et al., 2015 (57)	269	3.0	I	DFS, CSS
4	2b	Yang et al., 2018 (58)	98	2.22	I-IV	OS, PFS
14.1	2b	Dell’Aquila et al., 2018 (59)	413	3.0	IV	OS

NLR, neutrophil-to-lymphocyte ratio; CRC, colorectal cancer; CSS, cancer-specific survival; OS, overall survival; DFS, disease-free survival; RFS, recurrence-free survival; PFS, progression-free survival.

### Prognostic Value of Post-Treatment NLR in CRC

Compared to pre-treatment NLR, post-treatment NLR is much less investigated; however, the prognostic value of post-treatment NLR may be as important as that of pre-treatment NLR. Shibutani et al. demonstrated that increased post-operative NLR was an independent predictor for a poor OS in 254 patients with stage II/III CRC who underwent curative resection and oral fluorouracil monotherapy. Furthermore, patients with high post-operative NLR had a significantly shorter OS than those with low post-operative NLR in the low pre-operative NLR subgroup (63). Similarly, Hayama et al. reported that increased post-operative day 7 NLR was an independent prognostic factor of decreased recurrence-free survival (RFS) for stage II CRC in 176 individuals receiving curative surgery, and the study concluded that such patients may be candidates for adjuvant chemotherapy (64). Finally, Guo et al. showed that elevated pre-operative along with larger pre-operative to post-operative change in NLR had a significant correlation with poor OS but not DFS in 135 patients with stage I-IV CRC without significant correlation with DFS (65) (**Table 2**).

**Table 2:** Studies of prognostic of post-treatment NLR in CRC.

Impact Factor	Evidence Level	Study	Sample size	Cut-off value of NLR	TNM stage	Negative impact on survival
1.96	2b	Shibutani et al., 2015 (63)	254	2.5	II-III	OS
2.6	2b	Hayama et al., 2020 (64)	176	3.1 (post-operative 7th day)	II	RFS
2.2	2b	Guo et al., 2018 (65)	135	0.037 (pre- to post-operative change)	I-IV	OS

NLR, neutrophil-to-lymphocyte ratio; CRC, colorectal cancer; OS, overall survival; RFS, recurrence-free survival.

## Anti-Inflammatory Nutritional Intervention

As mentioned above, cancer patients are at risk of SIR and malnutrition; hence, it is recommended to evaluate systemic inflammatory and nutritional status before cancer treatments. Oral nutritional supplements (ONS) or parenteral nutrition (PN) for those with inadequate enteral nutrition should be administered to meet the goal of 25–30 kcal/kg/day with 1.2–1.5 g protein/kg/day in patients at risk of malnutrition. Anti-inflammatory ingredients, such as n-3 PUFAs contained in fish oil, serve as a means of immune modulation for patients with cancer, who are at risk of SIR (9). Although other nutrients, including glutamine, arginine, curcumin, vitamin D3, vitamin B12, probiotics, selenium, zinc, and so on, also have anti-inflammatory properties, some of them have no effect on body weight and in order not to complicate the issue discussed, this review focused on n-3 PUFAs.

Apart from providing energy, n-3 PUFAs have an additional function in modulating lipid metabolism and SIR. N-3 PUFAs can be incorporated into phospholipids at cell membrane that may influence synthesis of secondary messengers and modulate the expression of certain adhesion molecules at the surface of lymphocytes, monocytes and endothelial cells (66, 67). Previously, n-3 PUFAs have been demonstrated to modify fluidity and permeability of cell membrane as well as to regulate cell membrane receptors and enzyme activities (68, 69). Additionally, n-3 PUFAs compete with n-6 PUFAs in the cyclooxygenase-2 (COX-2) pathway to produce anti-inflammatory leukotrienes, prostaglandins, and thromboxanes (70, 71), and thereby regulate the expression of pro- and anti-inflammatory cytokine genes (72). Thus, it appears to be a promising strategy in the context of conditions with inappropriate pro-inflammatory activity, such as major surgery (73–76), critical illness (77–79), and cancer (9).

The effects of n-3 PUFAs are dose and time dependent, and no correlation with clinical outcomes can be observed in a short period of perioperative use (80–82). Ranging from 8 weeks to 24 months according to individual studies, taking oral nutritional supplement (ONS) containing fish oil or n-3 PUFAs has been shown to lower inflammatory biomarkers and improve SIR in CRC patients (83–85). Moreover, taking ONS containing fish oil EPA or n-3 PUFAs was also associated with improved QoL through reducing chemotherapy related adverse events, such as fatigue, diarrhea, loss of appetite and neuropathy (84, 86), and therefore physical function can be improved (87, 88). Song et al. demonstrated that a higher intake of n-3 PUFAs (0.35 g/day) was associated with a lower risk of CRC with high FOXP3^+^ tumor infiltrating T cells, which regulate the development and function of regulatory T cells to shut down the immune response to cancer cells (89). Kansal et al. revealed fish oil suppressed cell growth and metastatic potential by regulating PTEN and NF-κB signaling in colorectal cancer (90). As a result, n-3 PUFAs are supposed to have a positive impact on cancer prognosis; however, the related clinical evidence is inconsistent according to the methodology, including duration and amount of intake of n-3 PUFAs, patients’ cancer stage, and nutritional and systemic inflammatory status as well. One cohort study enrolled 1659 patients with stage I-IV CRC showed that a higher n-3 PUFAs consumption by increased ≧ 0.15 g/day after diagnosis was associated with a better CSS compared with intake by increased < 0.02 g/day or no change. No association was found with OS (91). One study showed no differences in recurrence or survival in stage II CRC across the 24-month follow-up (87), and another randomized clinical trial showed that taking 2 g/day of fish oil- EPA containing 0.6 g/day of eicosapentaenoic acid (EPA) and docosahexaenoic acid (DHA) during or after chemotherapy was associated with a longer PFS despite a lack of statistical significance in stage II-IV CRC (92). Notably, Shirai et al. demonstrated fish oil-enriched ONS containing 1.1 g of EPA and 0.5 g of DHA per day for 6 months was not only significantly associated with increased skeletal muscle and lean body mass and improved tolerance to chemotherapy, but also with a longer OS in gastrointestinal cancer patients with a modified GPS score of 1 or 2 (93). To take inflammatory and nutritional status into consideration, n-3 PUFAs may benefit selected cancer patients. Nevertheless, it is difficult to address the exact adequate amount of n-3 PUFAs supplement in cancer patients. According to Academy of Nutrition and Dietetics, which summarized nine studies (four randomized controlled trials, two non- randomized controlled trials, prospective cohort studies, and one time-series study), consumption 1.2 g to 2.2 g of EPA per day resulted in weight gain or weight stabilization in adult oncology patients with weight loss (94–102). In sum, it is reasonable to take at least 2.2 g of EPA per day for both inflammatory and nutritional purposes. More studies are needed to confirm the oncological outcomes of anti-inflammatory nutritional intervention of n-3 PUFAs.

## Consensus Recommendations

### Cut-Off Value of NLR

Studies had provided evidence of the prognostic and predictive value of elevated NLR in pre-treatment or post-treatment of mCRC. It is difficult to define a definite cut-off value of NLR prospectively, because all cut-off values reported were calculated retrospectively. Despite the threshold for NLR varied based on the statistical significance of cut-off value in each trial, the most common threshold for NLR was ≥3 (41), which is also the recommended value in this consensus [Grading of Recommendations, Assessment, Development, and Evaluation (GRADE): moderate].

### Anti-Inflammatory Nutritional Intervention in Patients With CRC Before Treatment

**Figure 2:**
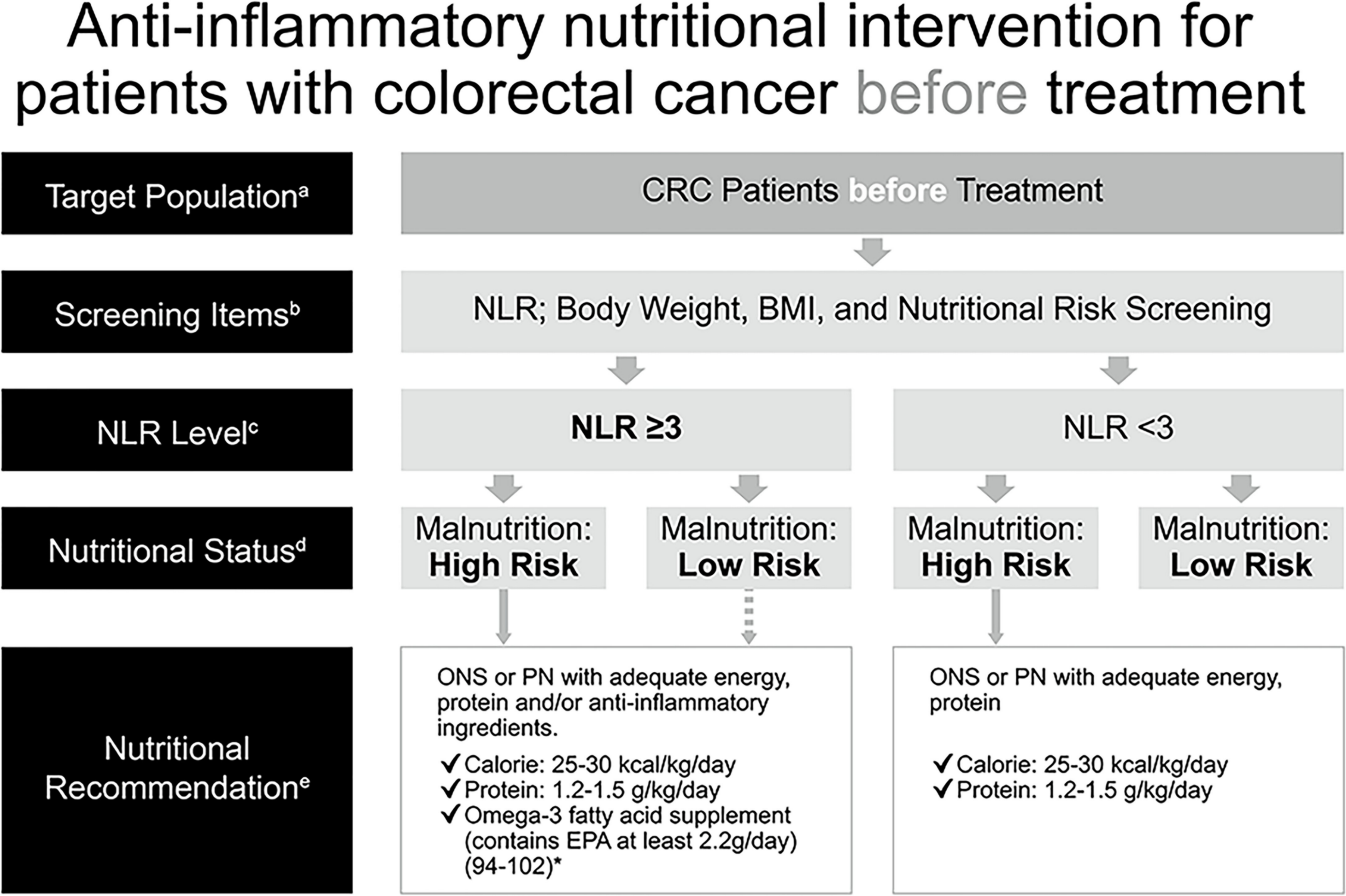
Anti-inflammatory nutritional intervention for patients with colorectal cancer before treatment. *(References 94–102) ^a^CRC patients before treatment (cancer-related drug treatment, radiotherapy and surgical treatment). ^b^Commonly used clinical nutrition screening/evaluation tools in Taiwan, which include PG-SGA, NRS 2002, MUST, MST. ^c^The NLR test is used to assess the risk of inflammation. ^d^Interpretation based on the evaluation/screening tools used in the nutrition evaluation clinical practice routines of each hospital. ^e^Based on the recommendations of ESPEN 2017, ASPEN, and the Academy of Nutrition and Dietetics, for cancer, diseases, and treatments that may cause inflammation, insufficient calorie intake, fatigue and low physical activity, omega-3 fatty acids (such as fish oil, EPA) should be consumed for their anti-inflammatory effects, in addition to ONS which provides additional calories and protein. Nutritional intervention can be performed if the patient has a high risk of malnutrition. There is no need to distinguish between precachexia and cachexia–directly start supplementing calories, protein, and omega-3 fatty acids for nutritional intervention, according to clinical guidelines, without further screening. The dotted line: If the patient is not at high risk of malnutrition but has an NLR ≥3, the follow-up decision depends on the physician, and the physician (or a supervisor) will then call the nutritionist. ASPEN, American Society for Parenteral and Enteral Nutrition; BMI, body mass index; CRC, colorectal cancer; EPA, eicosapentaenoic acid; ESPEN, European Society for Parenteral and Enteral Nutrition; MST, Malnutrition Screening Tool; MUST, Malnutrition Universal Screening Tool; NG, nasogastric; NLR, neutrophil-to-lymphocyte ratio; NRS, Nutritional Risk Screening; ONS, oral nutritional supplement; PG-SGA, Patient-Generated Subjective Global Assessment; PN, parenteral nutrition.

Malnutrition has a negative impact on clinical outcomes in patients with cancer and was associated with more post-operative infections (103–106) and complications (107, 108), reduced QoL (109), increased toxicities of chemotherapy leading to reduced dose or delayed treatment (5, 110), a shorter survival (103, 105–107, 111, 112), as well as a longer length of hospital stay (2, 103, 107, 108, 112), and therefore increased health care costs (2, 113). As a result, malnutrition should be corrected before treatment to prevent the aforementioned negative effects. Additionally, SIR, indicated by NLR elevation in this consensus, also correlated with poor oncological outcomes, especially in patients with advanced colorectal cancer (24, 51–55). In order to employ an appropriate nutritional intervention for patients with CRC, assessment for SIR with measurement of NLR (GRADE: moderate) and evaluation for nutritional risk with body weight, body mass index, and additional screening tool(s), which may depend on what is adopted by the individual institute in TSCRS (GRADE: high), are recommended to be made before treatment for all patients. Treatment includes surgery, chemotherapy, targeted therapy, radiotherapy, immunotherapy, or any kind of therapy against CRC. For patients with a high risk of malnutrition, ONS or parenteral nutrition (PN) for those whose enteral intake is inadequate should be administered with a target range of 25–30 kcal/kg/day and 1.2–1.5 g protein/kg/day to maintain or restore lean body mass (GRADE: high). ONS containing n-3 PUFAs (e.g. EPA) or lipid emulsion in PN should be added for patients with NLR ≥3 (GRADE: very low) (**Figure 2**). Obesity is a potential source of inflammation, which also correlates with NLR (114–117), and obese patients with CRC may at risk of malnutrition, therefore, the recommendations are applied to this population as well.

### Anti-Inflammatory Nutritional Intervention in Patients With Colorectal Cancer After Treatment

**Figure 3:**
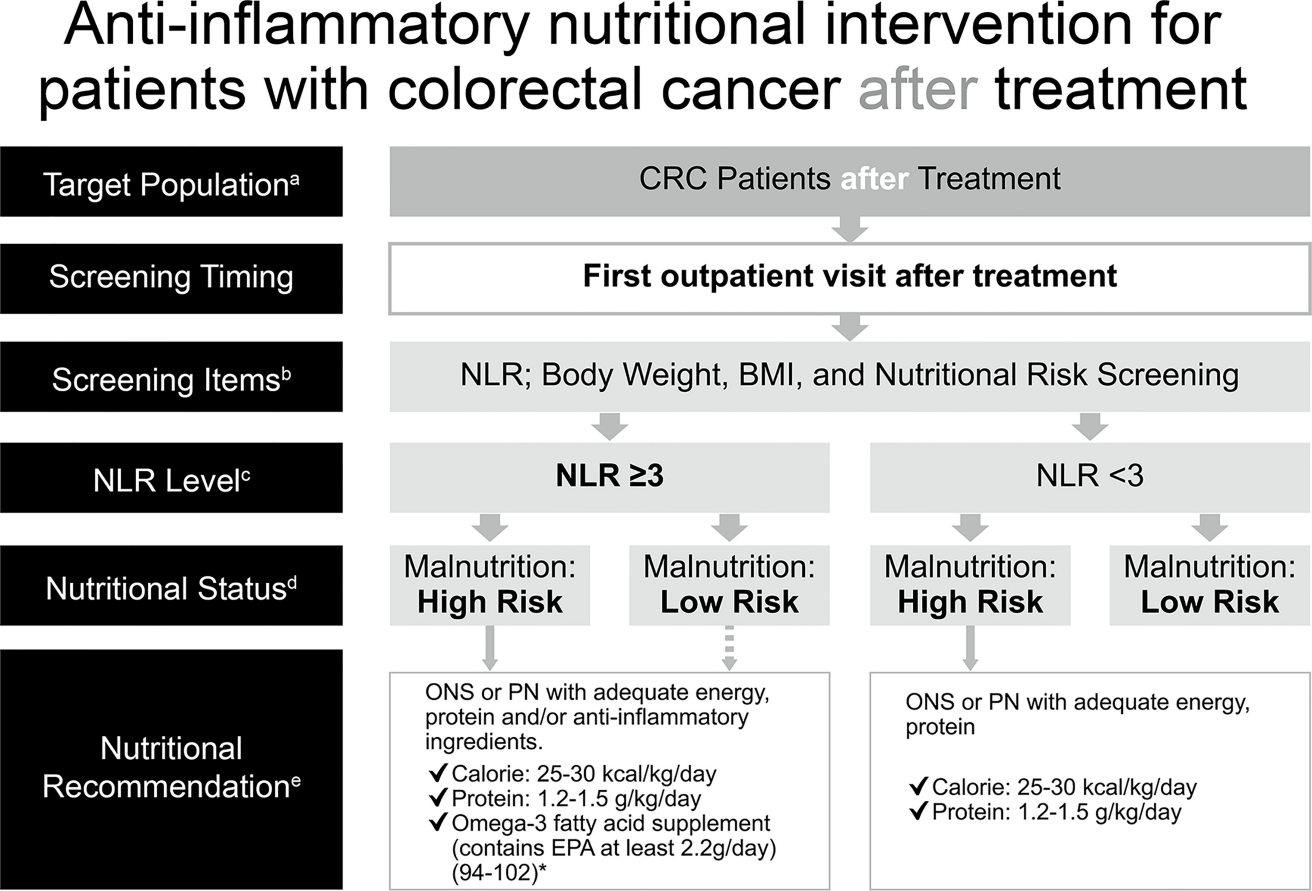
Anti-inflammatory nutritional intervention for patients with colorectal cancer after treatment. *(References 94–102). ^a^CRC patients post-treatment (surgery, chemotherapy, including adjuvant chemotherapy or palliative chemotherapy). ^b^Commonly used clinical nutrition screening/evaluation tools in Taiwan, which include PG-SGA, NRS 2002, MUST, MST. ^c^For patients with CRC after undergoing surgical treatment, the time point for postoperative NLR assessment is based on the clinical practice routines of blood sampling and tracking in various hospitals and on computer system settings. It is not strictly limited to 7 days; the first outpatient clinic after surgery is recommended. ^d^Interpretation based on the evaluation/screening tools used in the nutrition evaluation clinical practice routines of each hospital. ^e^Based on the recommendations of ESPEN 2017, ASPEN, and the Academy of Nutrition and Dietetics, for cancer, diseases, and treatments that may cause inflammation, insufficient calorie intake, fatigue and low physical activity, omega-3 fatty acids (such as fish oil, EPA) should be consumed for their anti-inflammatory effects, in addition to ONS which provides additional calories and protein. Nutritional intervention can be performed if the patient has a high risk of malnutrition. There is no need to distinguish between precachexia and cachexia–directly start supplementing calories, protein, and omega-3 fatty acids for nutritional intervention, according to clinical guidelines, without further screening. The dotted line: If the patient is not at high risk of malnutrition but has an NLR ≥3, the follow-up decision depends on the physician, and the physician (or a supervisor) will then call the nutritionist. ASPEN, American Society for Parenteral and Enteral Nutrition; BMI, body mass index; CRC, colorectal cancer; EPA, eicosapentaenoic acid; ESPEN, European Society for Parenteral and Enteral Nutrition; MST, Malnutrition Screening Tool; MUST, Malnutrition Universal Screening Tool; NLR, neutrophil-to-lymphocyte ratio; NRS, Nutritional Risk Screening; ONS, oral nutritional supplement; PG-SGA, Patient-Generated Subjective Global Assessment; PN, parenteral nutrition.

Cancer patients may still be at risk of malnutrition after treatment at discharge because nutritional status is dynamic and may decline after treatment owing to additional SIR and stress metabolism induced by treatments for cancer. It was reported that 36.4% of cancer patients were at nutritional risk at discharge and only one-third of patients at risk of malnutrition had received any kind of nutritional intervention at discharge (2). Moreover, high post-treatment NLR was negatively associated with oncological survival (63–65). Of note, the immunomodulatory effects of n-3 PUFAs that reduce SIR are dose- and time-dependent and take at least 8 weeks to occur (83–85). Consequently, analyzing nutritional risk and SIR after treatment allows the implementation of appropriate nutrition therapy. Based on availability and convenience of clinical practice, the current consensus recommends checking NLR (GRADE: low) and nutritional risk with body weight, body mass index, and other appropriate screening tools at the first outpatient visit (GRADE: moderate). Likewise, ONS or PN of isocaloric 25–30 kcal/kg/day and isonitrogenous 1.2–1.5 g protein/kg/day is recommended to be administered as nutritional support (GRADE: high), and additional n-3 PUFAs should be employed for patients with NLR ≥3 (GRADE: very low) (**Figure 3**).

## Conclusions

NLR is an economic and easily applicable inflammatory marker that predicts outcomes of cancers. Assessments for SIR and nutritional risk should be carried out before and after treatment. Accordingly, anti-inflammatory nutritional intervention not only provides energy but also modulates inflammatory response for patients at risk of malnutrition and SIR to facilitate optimal outcomes.

## Limitations and Strengths of This Consensus

The current consensus is confined to the available resources, the available therapeutic options in the individual institute in TSCRS, and the associated rules and laws of Taiwan National Health Insurance Administration, Ministry of Health and Welfare specifying the current Taiwanese clinical practices. It is not a dogmatic management guideline but should be adapted according to local circumstances of the individual institution in TSCRS. The consensus may need updating; for example, the cut-off value of NLR may be adjusted with more real world evidence gathered and analyzed by TSCRS in the future. The strength of this consensus is that it provides a guide, rather a rule to manage CRC patients at nutritional risk with or without SIR. Lastly, the proposed recommendations are relevant to local clinical practice adopted in Taiwan.

## Author Contributions

C-JM wrote the original draft. C-JM, M-CH, J-MC, P-SH, H-SW, C-LC, H-MH, C-CC, and J-YW reviewed and discussed the literature. J-YW reviewed and edited the manuscript. All authors have read and approved the submitted version of manuscript.

## Funding

This work was supported by grants through funding from the Ministry of Science and Technology (MOST 109-2314-B-037-035, MOST 109-2314-B-037-040, MOST 109-2314-B-037-046-MY3, MOST110-2314-B-037-097) and the Ministry of Health and Welfare (MOHW109-TDU-B-212-134026, MOHW109-TDU-B-212-114006, MOHW110-TDU-B-212-1140026) and funded by the health and welfare surcharge of on tobacco products, and the Kaohsiung Medical University Hospital (KMUH110-0R37, KMUH110-0R38, KMUH110-0M34, KMUH110-0M35, KMUH110-0M36, KMUHSA11013, KMUH-DK(C)110010, KMUH-DK(B)110004-3) and KMU Center for Cancer Research (KMU-TC109A04-1) and KMU Center for Liquid Biopsy and Cohort Research Center Grant (KMU-TC109B05) and KMU Office for Industry-Academic Collaboration (S109036), Kaohsiung Medical University. In addition, this study was supported by the Grant of Taiwan Precision Medicine Initiative, Academia Sinica, Taiwan, R.O.C.

## Conflict of Interest

The authors declare that the research was conducted in the absence of any commercial or financial relationships that could be construed as a potential conflict of interest.

## Publisher’s Note

All claims expressed in this article are solely those of the authors and do not necessarily represent those of their affiliated organizations, or those of the publisher, the editors and the reviewers. Any product that may be evaluated in this article, or claim that may be made by its manufacturer, is not guaranteed or endorsed by the publisher.
